# Description of an Advantageous Optical Label-Free Biosensing Interferometric Read-Out Method to Measure Biological Species

**DOI:** 10.3390/s140203675

**Published:** 2014-02-21

**Authors:** Miguel Holgado, Francisco J. Sanza, Ana López, Álvaro Lavín, Rafael Casquel, María F. Laguna

**Affiliations:** 1 Micro-nano Photonics and Biophotonics Group, Centro Láser and Centro de Tecnología Biomédica, Universidad Politécnica de Madrid, Av. Ramiro de Maeztu 7, 28040 Madrid, Spain; 2 Applied Physics Department, Escuela Técnica Superior de Ingenieros Industriales, Universidad Politécnica de Madrid, José Gutiérrez Abascal 2, 28006 Madrid, Spain

**Keywords:** photonic biosensors, label-free, rotavirus, optical read-out, environmental monitoring, health monitoring

## Abstract

In this article we report a new, simple, and reliable optical read-out detection method able to assess Rotavirus present in human sera as well as in the viral pollution sources. It is based on the interference of two interferometers used as biophotonic transducers. The method significantly improves the optical label-free biosensing response measuring both, the concentration of the AgR and its corresponding size. Two different immunoassays were carried out: Bovine Serum Albumin (BSA), and the recognition by its antibody (anti-BSA); and Rotavirus (AgR) and the recognition by its antibody (anti-AgR). In the cases studied, and using as model interferometer a simple Fabry-Perot transducer, we demonstrate a biosensing enhancement of two orders of magnitude in the Limit of Detection (LoD). In fact, this read-out optical method may have significant implications to enhance other optical label-free photonic transducers reported in the scientific literature.

## 1. Introduction

A biosensor is a device to detect specific biological species or chemical compounds. Among the different types of biosensors, label-free optical biosensors function without tags or chemical amplification. This optical direct *In-Vitro* Diagnostic (IVD), unlike labeled IVD, offers a direct detection of biomolecules accumulated or recognized on a given sensing surface. However, in the absence of this chemical amplification, it is a challenge for label-free biosensing to achieve the same degree of sensitivity as those exhibited by standard enzyme-linked immunosorbent assay (ELISA). In this sense, significant optical label-free biosensors are reported performing well. For example, several target biomolecules (e.g., DNA, Proteins, viruses, Bacteria or Cells) detected from different optical biosensors are well described and reviewed in recent articles [1–4].

Some of the most attractive examples are those based on: surface plasmon resonance [5,6] or ring disk resonators [7–9], Mach-Zehnder Interferometers [10], photonic crystals [11–14], Young interferometers [15], porous silicon [16,17], slot waveguide [18–21], BICELLs [22–24], among others [25,26].

What previous label-free optical biosensors share in common is the use of photonic architectures to produce resonant or interferometric optical modes suitable for enhancing the biosensing signal. When a biological component is immobilized (bioreceptor) or recognized (target biomolecule) on the sensing surface of a photonic transducer, optical resonances change producing variations in some interrogation magnitudes. Thus, the biosensing system depends not only on the photonic transducer proprieties, but also on the read-out optical method employed, as essential part to the optical sensing system as a whole. In most of the biosensors mentioned above, similar optical detection methods are employed for reading photonic transducer signals such as wavelength or angle variation of a resonant peak or dip, phase shift variation, or amplitude [27–29], or subtraction of wavelength-based optical signals are also well described in porous Si devices. The aim of this scientific report is not to compare different biophotonic transducers, but rather to describe in detail an advantageous optical read-out method for improving the LoD of any biophotonic transducer like those reported in the scientific literature.

In this article, we report an advantageous interferometric optical read-out method to enhance the biosensing response of a given photonic transducer measuring the accumulation (immobilization or recognition) of biological components and their corresponding biofilm thickness. We explain and demonstrate in detail how the biosensing response can be significantly enhanced, in comparison with the classical monitoring methods (e.g., the interfering peaks' or dips' wavelength displacements in a spectrometry profile), by operating with the interferometric optical sensitive signals coming from the transducer, and specifically converting them to optical power for a certain spectral range that depends of the biophotonic transducer type employed. In other words, we describe in detail how by measuring the optical power carefully selecting a specific spectral band, where the interfere intensity given by the optical transducer employed is higher, the LoD is drastically improved in comparison with those methods based on spectral change such as a shift in peak or dip location. Furthermore, this method can avoid common dispersive and complex optical elements such as gratings, high-numerical aperture objectives or costly interferometers, which reduce implementing costs, and makes the read-out system independent on the dispersive element resolution (e.g., wavelength, wavenumber or angle resolution).

Although in this article we explain in detail the theory involved and the experimental results for only a photonic transducer model based on a simple Fabry-Perot interferometer (FPI), what we find remarkable is that this optical read-out method can be employed for many other optical biosensors. In fact, we think that our findings reported in this article may have relevant implications for improving the majority of label-free biosensor reported in the literature. We also believe this method may be an effective approach for integrating the reading system and photonic transducers.

## 2. Experimental Section

The Interferometric Optical Detection Method (IODM) is characterized by the use of two interferometric signals, which allows for the optical reading system to convert the changes caused by the optical transduction into a unique, sensitive variable of detection. Therefore, two interferometric measurements are used: a first interferometric optical reference, (*I_Ref_^Interferometric^[wn_1_, wn_2_*]), which represents the measured intensity modulated by a reference interferometer (*IF_Ref_*); and a second interferometric signal measurement (*I_Out_^Interferometric^[wn_1_, wn_2_]*) observed in the sensing region of the signal interferometer (*IF_Out_*). The sensing surface region of *IF_Out_* is where changes produced by the biomolecular interaction takes place, for example, due to its functionalization by incorporating molecular receptors or because, already having incorporated molecular receptors, it has been used recognizing target molecules. The first and the second interferometric measurements can be taken sequentially or in parallel. If taken in parallel, the sensing observation region is physically different but it is interpreted with the same given the equivalence of the interferometric properties.

**Figure 1.**
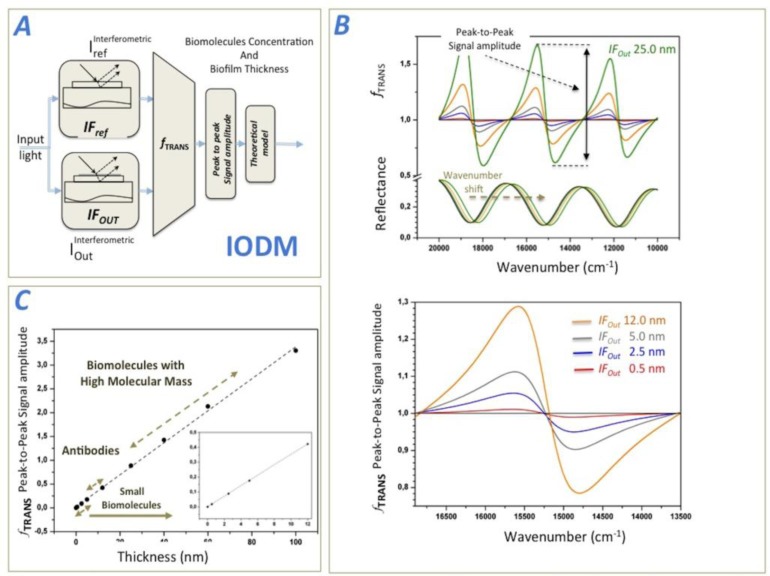
(**A**) Description of the Interferometric Optical Detection Method (IODM) particularized for a Fabry-Perot interferometer based on a SiO_2_ thin layer over Si as transducer model, (**B**) Optical simulation of the transduction function *f_TRANS_* coming from the reflectance optical response of *IF_Out_* for different biofilms thicknesses, (**C**) Theoretical sensing response considering the peak-to peak signal amplitude of *f_TRANS_* as transducing signal. It is also shown the different size of expected biomolecules to be detected.

A transduction function (*f_TRANS_*) is then constructed from the interferometric measurements, and analyzed to determine the biosensing response caused by the biological accumulation in the sensing observation region. Figure 1A shows a schematic representation of the IODM, where an input light is modulated by the two above-mentioned interferometers *IF_Ref_* and *IF_Out_*. *IF_Ref_* generates the interferometric reference and *IF_Out_* produces the interferometric signal coming from the biological accumulation. *f_TRANS_* is the operation between both interferometric signals and delivers an unique biosensing variable.

Though, useful transduction functions results from *IF_Out_* and *IF_Ref_* interferometric signals, and *IF_Out_* and *IF_Ref_* may be any of the aforementioned photonic transducers, for simplicity in this article to demonstrate the concept we use: a FPI as photonic transducers model consisting of a thin layer of SiO_2_ of 1,012 nm in thickness over a Si and a common transduction function *f_TRANS_* resulting from a quotient (see Equation (1)), which lead to a reliable detection function for a given wavenumber range from *wn_1_* to *wn_2_*:

(1) $$f_{\text{TRANS}}\;[wn_{1},wn_{2}]={I_{\text{Out}}}^{\text{Interferometric}}[wn_{1},wn_{2}]/{I_{\text{Ref}}}^{\text{Interferometric}}[wn_{1},wn_{2}]$$

Figure 1B shows the theoretical, modulated interferometric reflectance signals and how they operate by mean of *f_TRANS_* to produce a detection function between 20,000 and 10,000 cm^−1^. After having transformed the output light beams into numerical values, the transduction function can be mathematically represented (e.g., the wavenumber-dependent intensity or relative optical power). Thus, the transduction function, *f_TRANS_* measures the degree of biomolecular accumulation (biosensing response) in the observation region of the signal interferometer *IF_Out_*. Literature describing the reflectance output signal of an interferometer formed by a film layer of SiO_2_ is easily found [24,30]. Later in this paper, we compare these theoretical predictions with experimental results by measuring different types of biomolecules. When biomolecules are not present in the sensing area of *IF_Out_*, the output interferometric signals of *IF_Ref_* and *IF_Out_* are still the same, leading to an *f_TRANS_* equal to one for the wavenumber range considered. However, if there is a small accumulation of biomolecules in the sensing area of *IF_Out_*, *f_TRANS_* changes significantly. For this theoretical simulation, we modeled biofilms ranging in thickness from 0.5 nm to 60 nm in order to simulate varying sizes of biomolecules (e.g., a coating is roughly 2.5 nm for BSA, 14 nm for anti-BSA, and 60 nm for a virus).

The theoretical *f_TRANS_* behavior is shown in Figure 1B. To establish a single *f_TRANS_* measurement parameter to determine the degree of biological accumulation change in the sensing area, several options can be used:

1. A peak amplitude of function *f_TRANS_*,
2. A peak-to-peak amplitude of function *f_TRANS_*,
3. The change in slope in function *f_TRANS_* by a pre-established wavenumber value,
4. Considering *I_Out_* and *I_Ref_* of *f_TRANS_* as their corresponding optical power (or irradiance) for a given wavenumber range *[wn_a_, wn_b_].*

For this paper, we analyzed Option 2—A peak-to-peak amplitude of function *f_TRANS_*, in order to explain the IODM in a descriptive manner as proof of concept; and Option 4—Considering *I_Out_* and *I_Ref_* of *f_TRANS_* as their corresponding optical power, for enhancing the biosensing response of a given photonic transducer.

Although options 1 to 3 can be used for explain the optical read-out method, due to the fact *f_TRANS_* is constructed with interferometric signals coming by the biophotonic transducers, it must be remarked here that only option 4 enhances the LoD of the biosensing system. Thus, the novelty for enhancing the LoD is in choosing the appropriate wavenumber range for a given *f_TRANS_* where to measure the optical power. This wavenumber band will depend mainly of the type of interferometric signals *I_Out_* and *I_Ref_* produced for the biophotonic transducers employed for the biosensing. Even more, if a wrong wavenumber range or large spectral band is chosen, the LoD not only does not improve, but the possibility of no sensing for the system at all also exists. However, we consider it appropriate to describe also option 2 for a better understanding of the optical sensing mechanism and then explain in detail option for subject of this article as a main novelty.

Option 2: Figure 1C illustrates the theoretical biosensing response of the method for the peak-to-peak signal amplitude of *f_TRANS_* between 17,000 and 14,000 cm^−1^, and the different observable, accumulated, biofilm thicknesses in the *IF_Out_*. It can be observed a lineal response of this signal as the biofilm thickness increases in *IF_Out_*. For this calculation, we considered a biofilm refractive index of 1.4 [31].

Option 4: we also studied the variation of *f_TRANS_* considering the *I_Out_*, and *I_Ref_* (Equation (1)) as their corresponding *P_Ou_*_t_ and *P_Ref_* (optical power or irradiance) from *IF_Out_* and *IF_Ref_* respectively, and for a given wavenumber range (*wn_a_, wn_b_*). The objective is to discover the wavenumber range (*wn_a_, wn_b_*) where to measure *P_out_* and *P_Ref_* of the interferometric signals *IF_Out_* and *IF_Ref_* of *f_TRANS_* to significantly enhance the LoD.

**Figure 2.**
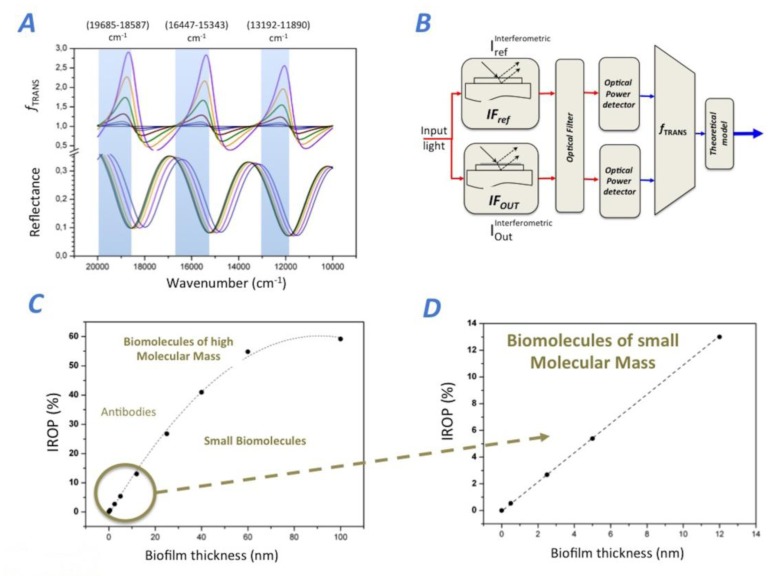
(**A**) Interferometric Optical Detection Method (IODM) considering *I_Out_* and *I_Ref_* of *f_TRANS_* as their corresponding Optical Power (*P_Out_, P_Ref_*) for a given wavenumber range [*wn_a_, wn_b_*] highlighted in blue. (**B**) Description of IODM particularized for a given wavenumber. (**C**) Theoretical response of the Increased Relative Optical Power (IROP) as a function of the biofilm thickness and (**D**) a detail of the IROP for molecules of small molecular mass.

We observe three spectral ranges where the optical power rises as the biofilm thickness increases specifically for the photonic transducer considered in this article (see Figure 2A). The observation wavenumber ranges (*wn_a_, wn_b_*) are: (13,192.6, 11,890.6) cm^−1^, (16,447.4, 15,243.9) cm^−1^ and (9,685.0, 18,587.4) cm^−1^. In order to simulate *f_TRANS_* for this case, we have to obtain the optical power of the *IF_Ref_* and *IF_Out_* for one of these wavenumber ranges considered. Thus *f_TRANS_* for Option 4, can be calculated as the integral of reflectance signal of *IF_Ref_* (*P_Ref_*) divided by the integral of reflectance of the *IF_Out_* (*P_Out_*) for the particular wavenumber range (*wn_a_, wn_b_*). For this case, *f_TRANS_* directly represents the relative optical power, which is directly correlated to the biofilm thickness accumulated in the *IF_Out_* sensing surface. As abovementioned, in order to produce the best biosensing response, it is critical to select the proper wavenumber range (*wn_a_, wn_b_*) where *f_TRANS_* produces a maximum of relative optical power.

Once the suitable location of the wavenumber range is selected, the transduction function *f_TRANS_* makes it possible to assess the value of the increased relative power as a function of the biofilm thickness. A proper wavenumber range with a good signal-to-noise ratio (SNR) in our optical set-up is (13,192.6, 11,890.6) cm^−1^(from 758 to 841 nm), where *f_TRANS_* amplitude rises when the biofilm thickness increases. Therefore, the increased relative optical power (IROP) is a function of the biofilm thickness accumulated in the *IF_Out_*, and this IROP can be calculated (see Equation (2)):

(2) $$\text{IROP}[\%]=(f_{\text{TRANS}}-1)\times 100=((P_{\text{Out}}[wn_{a},wn_{b}]/P_{\text{Ref}}[wn_{a},wn_{b}])-1)\times 100$$

Therefore, the IODM is the IROP caused by the interference of the two interferometers *IF_Ref_* and *IF_Out_*. It is important to remark here that the chosen wavenumber range optimizes the sensitivity of the biosensing system (defined as the variation of the IROP as a function of the biofilm thickness) for the best SNR, and therefore the manner in which we can improve significantly the LoD. Figure 2B shows a schematic representation of the IODM for option 4 for the model FPI considered. The method for Option 4 directly obtains the biosensing curve just by reading the optical power of each interferometer. Figure 2C,D shows the biosensing response IROP (Equation 2) as a function of the biofilm thickness accumulated onto the *IF_Out_*. A linear response for biomolecules smaller than 60 nm is observed. In fact, the theoretical biosensing sensitivity (measured as the slope of the sensing curve) is 0.975% nm^−1^, and it is lower for biomolecules higher of 40 nm (see Figure 2C).

### 2.1. Interferometers

**Figure 3.**
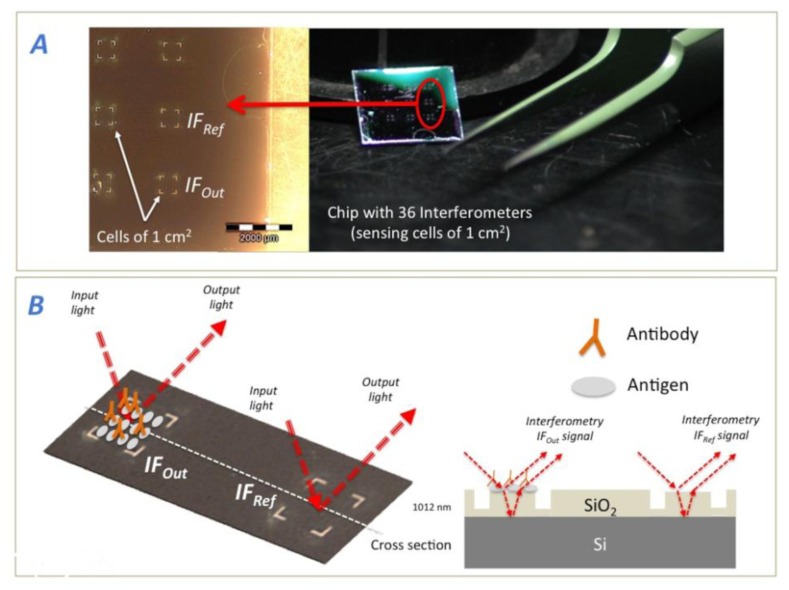
(**A**) Optical image of a chip with 36 FPIs. (**B**) A schematic representation of the Biosensing process for the IODM, where a reference interferometer *IF_Ref_* and a signal Interferometers *IF*_Out_ are used.

Interferometers were fabricated by thermal oxidation on a silicon wafer reaching a SiO_2_ thickness of 1,012 nm. We employed a laser workstation to define the sensing areas by direct writing ablation (see Figure 3). The tool is a nanosecond regime UV laser (Nd:YVO_4_) at 355 nm using a third harmonic generator. Thus, simple 1 cm^2^ interferometers as sensing sites were defined as *IF_Ref_* and *IF_Out_* to carry out the experiments. After fabrication, there is an ultrasonic cleaning of the chip embedded in ethanol, and a second process of the biochips through a “piranha solution” consisting on a reaction of H_2_SO_4_+H_2_O_2_ on a stoichiometric ratio 2:1. This ensures the elimination of all dirty or biological material on the chips surface and facilitates the molecules attachment. Besides; it allows a proper signal measurement with the detector.

### 2.2. BSA/ Anti-BSA Immunoassay

BSA coating was performed by immobilizing by dropping 50 μL of a 100 μg/mL concentration solution and incubating it for 30 min at 37 °C under humidity conditions to prevent evaporation. In order to achieve the best condition for a physical adsorption onto the *IF_Out_* SiO_2_ sensing surface BSA solutions were prepared at pH 5 due to the fact that the isoelectric point of BSA is around pH 4.7 [32]. Then, the *IF_Out_* surface was rinsed with (DI)-H_2_O and blown-dry with clean (particle-free) air. For anti-BSA recognition, concentrations were increased from 0 to 100 μg/mL by dropping of 50 μL onto the chip for 40 min in PBS solutions at 37 °C under humidity conditions to prevent evaporation. After each incubation/washing step, the surfaces were also washed with (DI)-H_2_O and blown-dry with clean (particle-less) air to eliminate unbounded molecules after incubation.

### 2.3. Rotavirus/Anti-Rotavirus Immunoassay

To reliably immobilize the AgR, we first covered the *IF_Out_* surface with a thin, suitable, stable layer of SU-8. For this coating it was used SU-8 2000.5 resist diluted 1:10 in cyclopentanone. After the SU-8 deposition, the chip was soft-baked at 70 °C for 1 min. Then, a UV light exposure is carried out in order to obtain a stable SU-8 layer on the sensing surface of the *IF_Out_*, followed by a post-bake at 70 °C for 5 min. Finally, to increase the hydrophilicity of the SU-8 an acid treatment was carried out. Thus, by partially open the SU-8 epoxy groups after treating the SU-8 surface by immersion in 95% sulfuric acid for 10 s, plus a wash in deionized water (DI)-H_2_O at room temperature, a highly hydrophilic surface was achieved, facilitating covalent bonds between the polymer and the bioreceptors [23]. Anti-AgR recognition was carried out by incubating increasing concentrations for 40 min at 37 °C following the same steps used for BSA/anti-BSA.

### 2.4. Optical Characterization

Interferometers, *IF_Ref_* and *IF_Out_* were optically characterized by means of Fourier Transform Visible and Infrared spectrometry (FT-VIS-NIR) using a Bruker Vertex 70 (Bruker Optik GmbH: Bremen, Germany) instrument adapted for visible and near infrared spectral range. Edge apertures of the Hyperion 1,000 microscope (attached to the spectrometer) were adjusted to focus the light on the 1 cm^2^ sensing areas of the FPIs. The light covers a spectral range from visible to near infrared: 20,000 to 10,000 cm^−1^ (500 to 1,000 nm). The spectra were carried out with a wavenumber resolution 4 cm^−1^. One thousand scans were performed for the background signal, and 100 scans for each measurement to ensure enough SNR.

## 3. Results and Discussion

For all of the experiments, the sensing areas of *IF_Out_* and *IF_Ref_* were both square cells of 1 cm^2^ size and the FPI employed was formed by 1,012 nm of SiO_2_ over Si as substrate (see Figure 3). All measurements were taken with a FT-VIS-IR spectrometer whose spot size was adjusted by the edge apertures to the 1 cm^2^ of the sensing area.

### 3.1. BSA/Anti-BSA Biofilms Measurements

**Figure 4.**
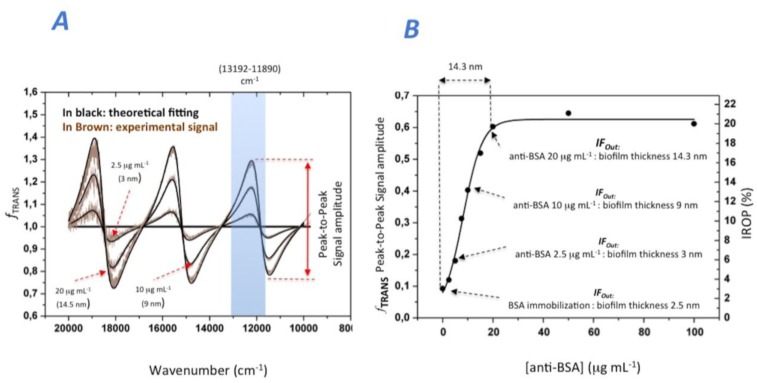
Experimental response for measuring BSA / Anti-BSA immunoassay: (**A**) *f_TRANS_* response where in brown is represented the experimental signal and in black the theoretical fitting of the model. (**B**) Biosensing response starting with the immobilization of BSA and the increasing concentrations of anti-BSA.

First, we immobilized the BSA onto the sensing surface of the *IF_Out_* interferometer (see Figure 4). In order to coat the entire sensing surface, a BSA concentration of 100 μg/mL was incubated. After this BSA coating, the biosensing recognition was examined by mean of incubating increasing concentrations of anti-BSA antibodies from 0 to 100 μg/mL. After each incubation and washing procedure, *f*_TRANS_ was obtained for different anti-BSA concentrations to obtain the biosensing response. Figure 4A shows the experimental *f*_TRANS_ for anti-BSA concentrations and Figure 4B the biosensing response. Figure 4B also shows the starting *f*_TRANS_ point that corresponds for the BSA saturation level. This value reaches 0.092 for peak-to-peak signal amplitude and 2.8% of IROP within the wavenumber range (13,192–11,890) cm^−1^. This BSA saturation point corresponds to a BSA biofilm thickness of about 2.6 nm (see Figure 1C), which is similar to those reported in the literature for a biofilm thickness of BSA [26].

The biosensing response for the different increasing anti-BSA concentrations was also studied in this experiment. A saturation of anti-BSA for 20 μg/mL for a *f_TRANS_* of 0.602 (peak-to-peak amplitude) and 19.7% IROP for the same wavenumber range (see Figure 4B) can be observed. This value indicates that a BSA plus anti-BSA biofilm thickness is in the order of 16.9 nm. Therefore, if we substrate the 2.6 nm of BSA, the anti-BSA biofilm thickness can be considered in the order of 14.3 nm, which is also similar to those previously reported [22]. Finally, the experimental sensitivity for this immunoassay is 1.2% of IROP per each μg/mL of anti-BSA.

### 3.2. Rotavirus/Anti-Rotavirus Biofilms Measurements

**Figure 5.**
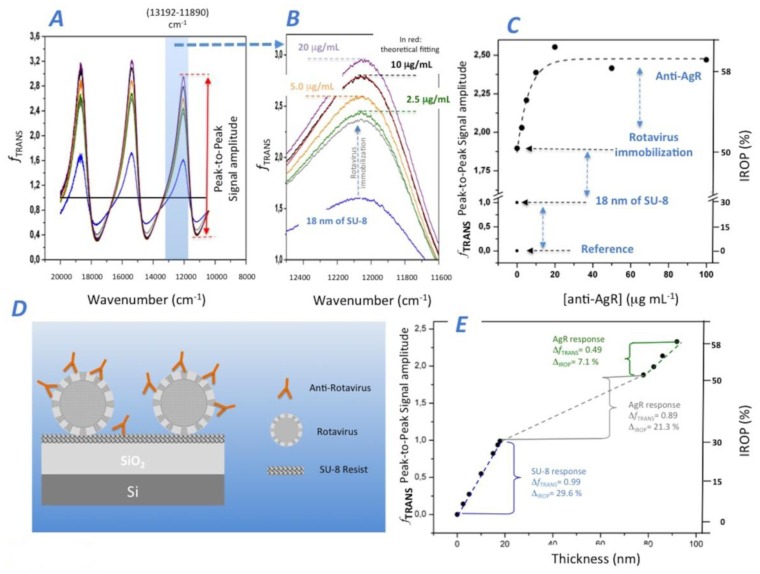
Experimental response for measuring Rotavirus/anti-Rotavirus immunoassay: (**A**) Experimental *f_TRANS_* obtained and (**B**) the detailed response within 13,192–11,890 cm^−1^. (**C**) Biosensing response for anti-AgR recognition. (**D**) Schematic representation of the experiment carried out and (**E**) theoretical biofilm thickness calculation.

To immobilize the AgR on the sensing surface, we first covered the *IF_Out_* sensing surface with a thin layer (18 nm). Second, a commercial rotavirus solution was applied to the SU-8 sensing surface of *IF_Out_*. After the AgR coating, we analyzed the IODM for the different increasing concentrations of anti-AgR antibodies from 0 to 100 μg/mL. Figure 5A shows the *f*_TRANS_ for the different experiment phases, from the SU-8 and AgR coating to the different increasing concentrations of anti-AgR, and Figure 5B shows in detail the obtained experimental response. The peak-to-peak amplitude and the IROP can be observed in detail in Figure 5C.

In order to analyze the experiment, we fit the experimental results to the theoretical model obtained for the different coatings steps onto the sensing surface of *IF_Out_*. It can be observed that the SU-8 coating produces a peak-to-peak signal amplitude of 0.99 and a 29.6% of IROP corresponding to a SU-8 thickness of 18 nm. Figure 5E shows the theoretical *f_TRANS_* response for different SU-8 biofilm thicknesses.

We analyzed the AgR coating on the SU-8, where an increase of peak-to-peak amplitude of 0.89 and 21.3% of IROP were obtained. The AgR size reported in the literature is between 50 and 70 nm in diameter [33]. We could consider a biofilm thickness of 60 nm covering completely the *IF_Out_*. For this case, the peak-to-peak an IROP values should reach 1.13 and 38.8% respectively, which are higher than the experimental results obtained for AgR coating. We assume that the lower values achieved for AgR immobilization are mainly due to a lower effective refractive index of 1.4 of the biofilm layer produced for the AgR immobilization. In fact, we cannot ensure that there was a perfect AgR monolayer on the sensing surface. Even so, a perfect triangular single layer array considering the spherical shape of viruses of 60 nm would produce a lower refractive index. The experiment results in a 1.2 refractive index for 60 nm of biofilm thickness.

Lastly, we analyzed the experimental biosensing response of the anti-AgR recognition (see Figure 5C). It was observed that saturation begins at a concentration greater than 10 μg/mL. At this level, the increased peak-to-peak amplitude and IROP are 0.49 and 7.1%, respectively. Even considering the complexity of the optical system (as it is shown in Figure 5D), the equivalent biofilm coating produced by anti-AgR antibodies can be calculated. By fitting the experimental results with the theoretical model for a refractive index of 1.4, the estimated coating results in a value of 14.1 nm, which still, seems to be quite reasonable for an antibody.

### 3.3. Improvement of the Limit of Detection by the IODM

The sensitivity (*m*) can be defined as the signal response to a particular biomolecule concentration, generally determined by the slope of the biosensing curve. A common method for estimating optical sensitivity is to consider, as signal response, the displacement of an interferometric (or resonant) optical mode (e.g., in wavelength, wavenumber or in angle of incidence) as a function of the target biomolecules concentration (or biofilm thickness covering the sensing surface).

The limit of detection is the smallest measured concentration that can be detected with reasonable certainty. An estimate of the LoD can be obtained by the quotient of the read-out signal uncertainty, and the sensitivity of the given transducer.

The most significant uncertainty sources are SNR and the resolution of the read-out optical system. For example, for a spectrometry-based system, this resolution is given in wavelength or wavenumber. By increasing the number of measurements, we can improve the SNR, while the resolution is normally fixed in the read-out optical system. Optical resolution used to be the primary factor of uncertainty when a high number of measurements are done for a specific concentration during the reading process.

In this article we evaluate how the IODM based on IROP enhances the LoD for the FPI transducer used as model, although – as above mentioned –, this method can be applied for other photonic transducers. The fourth option of IODM operates with the optical power of two interferometric modes: One used as reference and other used as signal, which allowed us to check the SNR during each biosensing stage. Thus, the changes caused by the biological accumulation in the optical power signal within a specific wavenumber range are converted in an increased of the relative optical power IROP (see Section 2). With the IODM based on IROP, the main uncertainty source to determine the LoD is due to the SNR of the optical power rather than by the wavelength or wavenumber resolution. Therefore, we must compare both optical methods: the common one based on the wavenumber shift variation and the IODM based on the IROP variation; for the FPI employed as model and for both immunoassays carried out.

For the first immunoassay, BSA/anti-BSA, the saturation point was reached for 20 μg·mL^−1^ and the dip wavenumber displacement at this point was 153.8 cm^−1^ (from 11,904.7 cm^-1^without anti-BSA to 11,750.8 cm^−1^ for 20 μg·mL^−1^ of anti-BSA) within the wavenumber range considered of the reflectance profile. Therefore, the wavenumber sensitivity (*m_wn_*) for this system can be calculated as 153.8 cm^−1^ divided by 20 μg·mL^−1^. As a result, *m_wn_* is 7.7 cm^−1^/ (μg·mL^−1^) for the FPI used as model. As all the measurements were carried out with a wavenumber resolution (*w_res_*) of 4 cm^−1^ and replicated 100 times for the signal and 1,000 times for the background (see Section 2.5); the most significant factor to calculate the measurement uncertainty is the wavenumber resolution. Thus, the expanded uncertainty (*U_wn_*) can be calculated multiplying the standard uncertainty (*u_wn_*) by the coverage factor (*K*) or confidence level. The International Union of Pure and Applied Chemistry (IUPAC) recommends use *K*=*3* to estimate the LoD, and the Guide to the Expression of Uncertainty in Measurements (GUM) [34] recommends calculate the standard uncertainty as the wavenumber resolution (4 cm^-1^ for this case) divided by the square root of 12: (*u_wn_*)^2^ = (*w_res_*)^2^/12. Thus, the uncertainty due to the spectrometer resolution *U_wn_* results in a value of 3.46 cm^−1^. For this expanded uncertainty the estimated LoD is 450 ng/mL^−1^, which is calculated by the quotient of (*U_wn_/m_wn_*). The value seems to be realistic for such a simple FPI transducer.

For the IODM subject to this article, the sensitivity (*m_IODM_*) achieved for the recognition of anti-BSA was 1.2% of IROP per each μg·mL^−1^ of anti-BSA. However, in this case LoD does not depend on the resolution of the spectrometer employed, but on the SNR. IODM produce an IROP signal even when there is not biomolecules accumulation on the sensing surface of *IF_Out_* being this signal fluctuation the noise level. For the same FPI and the same experimental conditions, the SNR was of 52.6 dB, leading a noise IROP signal of 5.48 × 10^−4^%. This SNR achieved is better than the 30 dB previously obtained because the area of the sensing area of the FPI transducer employed is much higher (1 × 1 cm) in comparison with the sensing area (60 × 60 μm) of the sensing sites previously used in that scientific report [22]. Thus, the uncertainty (*U_IODM_*) for this case can be estimated as three times the noise signal, reaching a value of 16.44 × 10^−4^%. Therefore, the estimation of the LoD is the quotient between 16.44 × 10^−4^% and 1.2%/(μg·mL^−1^) resulting in a LoD_IODM_ = 1.37 ng·mL^−1^. This value significantly improves in more than two orders of magnitude the LoD of 450 ng·mL^−1^ obtained by the common method using the same FPI transducer and for the same experimental conditions. Moreover, even by reducing the spectrometry uncertainty and measuring with a much higher resolution of 0.5 cm^−1^ (30 pm in wavelength for 12,000 cm^−1^), the common LoD is still 55.8 ng·mL^−1^, which is still much worse. Furthermore, measuring with higher resolution implies much more time to obtain the signal spectrum.

Finally, analyzing the anti-Rotavirus biosensing curve, we can obtain the experimental sensitivity: *m_IODM_* = 0.9% of IROP per each μg mL^−1^ of anti-AgR. This lower sensitivity, in comparison with the anti-BSA one previously obtained, can be explained theoretically due to the accumulated SU-8 and AgR thickness (see Figure 2C). The biosensing curve saturates at 10 μg·mL^−1^ and the IROP for this point is 7.1%. Therefore, the LoD for this immunoassay could be estimated as 16.44 × 10^−4^%/0.9%/(μg·mL^−1^), resulting in a value of 18.2 ng·mL^−1^, which is lower than the LoD obtained for anti-BSA recognition, but still really competitive for this simple FPI.

## 4. Conclusions

In this article we assess a new read-out IODM in an effort to significantly improve the LoD of a FPI used as photonic transducer model, and tested it for two different immunoassays. It is based on operation of the optical power of interferometric signals produced by a given transducer specifically for a spectral band, and not the LoD's dependence on the wavelength or wavenumber resolution of the spectrometer employed. Thus, we have demonstrated that this improvement is at least as good as two orders of magnitude for the same transducer and experimental conditions with this novel approach based on the IODM based on IROP if we select the optimum specific wavenumber range (or spectral band) to optimize sensitivity (IROP variation as a function of the biomolecules concentration) where the SNR is lower. This IODM is a viable and promising alternative to other biosensing methods reported in the scientific literature.

The theoretical predictions and experimental results demonstrate the advantages of using this optical biosensing read-out method for measuring biological species such as proteins and viruses, and estimating their biofilm thickness. With this IODM based on IROP we were capable of enhancing the LoD of a really simple photonic transducer based on a single layer of SiO_2_ from 450 ng·mL^−1^ to 1.37 ng·mL^−1^ recognizing anti-BSA, certifying the benefits of using this IODM. Moreover, we can calculate the anti-BSA and BSA biofilm thickness, achieving similar values to those reported in the literature, demonstrating the reliability of this method. Furthermore, we tested the IODM for a more complex experiment detecting easily SU-8 thin film deposited onto the *IF_Out_* sensing surface, the immobilization of the Rotavirus antigen, and the recognition of its corresponding antibody, achieving competitive LoD figures.

Finally, we can conclude that the IODM based on IROP is a promising approach for several reasons. First, IODM can function for other interferometric photonic transducers such as those based on ring or disk resonators, Mach-Zehnder interferometers, Young interferometers, BICELLs, and others reported for label-free biosensing. Secondly, the fact that the LoD does not depend on the resolution of costly optical dispersive elements such as gratings (wavelength), interferometers in the reader (wavenumber) or high numerical aperture objectives (angle); make IODM as a promising alternative for developing compact lab-on-a-chip devices, with plenty of room in the clinical settings.

It is expected that the LoD figures for other photonic transducers can be significantly enhanced according the results presented in this paper by measuring the IROP within the spectral band defined by the photonic transducer employed.
